# Case management to improve adherence for HIV-infected patients receiving antiretroviral therapy in Ethiopia: a micro-costing study

**DOI:** 10.1186/1478-7547-9-18

**Published:** 2011-12-20

**Authors:** Elliot A Marseille, Sebastian Kevany, Ismael Ahmed, Getachew Feleke, Bill Graham, Thomas Heller, James G Kahn, Michael Reyes

**Affiliations:** 1 Health Strategies International, 555 59th Street, Oakland, CA 94609 USA; 2 I-TECH, University of California San Francisco, Department of Family and Community Medicine, 50 Beale Street, Suite 1300, San Francisco, CA 94111 USA; 3 I-TECH, Addis Ababa, Ethiopia; 4 I-TECH, University of Washington, Department of Global Health, 901 Boren Avenue, Suite 1100, Seattle, Washington 98104, USA; 5 Institute for Health Policy Studies, University of California, San Francisco, 3333 California Street, Suite 265, San Francisco, CA 94118, USA

## Abstract

**Background:**

Adherence to antiretroviral medication regimens is essential to good clinical outcomes for HIV-infected patients. Little is known about the costs of case management (CM) designed to improve adherence for patients identified as being at risk for poor adherence in resource-constrained settings. This study analyzed the costs, outputs, unit costs and correlates of unit cost variation for CM services in 14 ART sites in Ethiopia from October 2008 through September 2009.

**Methods:**

This study applied standard micro-costing methods to identify the incremental costs of the CM program. We divided total CM-attributable costs by three output measures (patient-quarters of CM services delivered, number of patients served and successful patient exits) to derive three separate indices of unit costs. The relationships between unit costs and two operational factors (scale and service-volume to staff ratios) were quantified through bivariate analyses.

**Results:**

The CM program delivered 4,598 patient-quarters of services, serving 5,056 patients and 1,995 successful exits at a cost of $167,457 over 12 months, or $36 per patient-quarter, $33 per patient served and $84 per successful exit from the CM program. Among the 14 sites, mean costs were $11,961 (sd, $3,965) for the 12-month study period, and $51 (sd, $36) per patient-quarter; $48 (sd, $32) per patient served; and $183 (sd, $157) per successful exit. Unit costs varied inversely with scale (r, -0.70 for cost per patient-quarter versus patient-quarters of service) and with the service-volume to staff ratio (r, -0.68 for cost per patient-quarter versus staff per patient-quarter).

**Conclusions:**

For those receiving CM, the program adds 0.52% to the lifetime cost of ART. These data reflect wide variation in unit costs among the study sites and suggest that high patient volume may be a major determinant of CM program efficiency. The observed variations in unit costs also indicate that there may be opportunities to identify staffing patterns that increase overall program efficiency.

## Background

Over 1.1 million HIV-infected individuals reside in Ethiopia [1]. Adult HIV prevalence was estimated at 1.8% to 2.2% in 2009, low by the standards of sub-Saharan Africa [2]. Provision of free antiretroviral therapy (ART) started in October 2005, leveraging support from both the Global Fund to Fight AIDS, Tuberculosis and Malaria (GFATM) and the United States President's Emergency Plan for AIDS Relief (PEPFAR) [3]. Ethiopia is among PEPFAR's 15 most highly-funded "focus" countries. PEPFAR funding for ART scale-up rose rapidly over a 5 year period, from over $49 million in fiscal year (FY) 2004 to $354.5 million in FY 2008 [4]. Spending declined slightly to a planned $346 million in FY 2009. As a result of this investment, 163,100 patients received ART in 2009, country-wide. In the context of the current flat-lining of PEPFAR HIV funding [5], combined with substantial projected increases in the number of HIV-infected people who will be clinically eligible for treatment, the need to understand program costs and the trade-offs between various ways of structuring HIV treatment programs and adjunct services has never been greater. Case management (CM) to improve adherence, slow disease progression, lower costs and reduce the risk of the spread of treatment-resistant HIV virus to the community has received little attention.

The relationship between adherence to ART and favorable clinical outcomes is well established [6,7]. There is now also substantial evidence that good adherence may be associated with lower spending, likely due to improvements in the health status of adherent patients. Although the independent causal role of better adherence cannot be inferred from this study, a 2010 analysis of a South African managed care setting found that treatment and care costs were highest in the lowest quartile of adherence. Monthly costs in this lowest quartile were higher than quartiles 2, 3, and 4 by US$30, $59 and $85, respectively. The net savings reflected reductions in spending on opportunistic infections that exceeded the higher spending on ART [8]. The full social and economic benefits of greater adherence are underestimated by these results since they omit potentially improved labor productivity [9]. Other benefits include the subjective value of improved health to the patients and their families, and a lower risk to the community from the spread of resistant strains of HIV.

A non-randomized study in the United States found that individual case management for homeless HIV-infected clients improved adherence and CD4 counts [10]. We are unaware of similar studies in sub-Saharan Africa or other developing countries, though a simulation study set in Cote d'Ivoire found that adherence interventions (not including CM) are likely to be both effective and cost-effective in preventing loss to follow-up in resource-constrained settings [11]. The question of whether the cost of CM programs to improve adherence is justified by the benefits remains unanswered. The current study aims to provide part of the answer to that question by (1) estimating the unit costs of case management services for ART patients in three provinces in Ethiopia, (2) documenting the variation in those costs, and, (3) exploring the opportunities for greater efficiency in the provision of CM services.

## Methods

### Overview

The International Training and Education Center for Health (I-TECH) is a collaboration between the University of Washington and the University of California, San Francisco. It receives its principal funding from PEPFAR via the Health Resources and Services Administration and operates in 15 countries seeking to strengthen health delivery systems through training, evaluation and support for clinical services. In this study, we computed the monetized value of the resources consumed by CM activities carried out by I-TECH in cooperation with the Federal Ministry of Health of Ethiopia. We applied standard micro-costing methods in which each type of resource is quantified and assigned a unit cost [12]. We divided total CM-attributable costs by two output measures in order to derive two separate indices of unit costs, cost per patient-quarter of CM provided, and cost per successful exit from the program. The largest share of costs, those devoted to supporting the full-time staff providing direct CM services, was represented as an incremental cost of the CM program, that is the costs of CM activities entailed beyond ongoing ART operating costs. Overhead and administrative costs incurred at I-TECH headquarters in Addis Ababa were assigned to the CM program on an incremental basis where possible, and otherwise, according to the proportion that CM direct service staff constituted of total I-TECH direct service staff during the study period.

Historical salary and expenditure records were queried from QuickBooks, an electronic accounting system used to manage finances for the I-TECH program in Ethiopia. These expenditure data were verified with interviews of project managers, service delivery staff, and accounting staff. Costs were assessed from the perspective of the CM services provider. Time, costs or cost-savings from improved adherence, and other possible costs to patients were not assessed. All costs were converted from Ethiopian Birr to US dollars, at the exchange rate for March 31, 2009 (11.2 Birr/US $; http://www.oanda.com/currency/historical-rates/), the mid-point of the data period. Results are presented in 2009 dollars.

### Setting, intervention and study period

I-TECH provides support, including CM, to 46 health care sites in the three northern regions of Amhara, Afar, and Tigray, which account for nearly 50% of the national HIV burden. More than 241 I-TECH staff members facilitate government-sanctioned healthcare activities including training and capacity building, other health systems strengthening, and monitoring and evaluation. In 2006, at the request of the Federal HIV/AIDS Prevention and Control Office/Ministry of Health of Ethiopia, I-TECH Ethiopia and Visions for Development, Inc (VDF) developed a national model for case management for patients at risk of poor adherence. The program was validated and operationalized, first in six pilot hospitals and then successively expanded to a total of 46 sites. Case managers are trained using a national curriculum, and are high school graduates with modest experience in community health. We selected October 2008 - September 2009 as the study period since 12 months account for any seasonal caseload fluctuations, and administrative and compensation practices at all CM sites were the same during this period. Fourteen sites were selected for the current cost study, all of which had been in operation for at least 12 months; these included nearly a third of the sites with case management programs.

Patients at the ART clinic are referred by their ART clinicians to CM services based on presenting adherence risk factors, such as alcohol or *khat *(stimulant) dependency, lack of disclosure of HIV status to family members, lack of basic needs such as food, shelter and transportation, and disease severity or co-morbidity. The case management services consist of adherence counseling and support; health education; peer support; and referral of clients to community based organizations equipped to address specific barriers to adherence such as malnutrition, substance abuse or material needs for clothing, rent, and food.

### Client characteristics and continuity of care at baseline

A convenience sample survey of ART patients was conducted between May and June of 2007 (n = 60) in the six original sites chosen to pilot the case management program. It found that 85% were aged 20-45 and 15% were 46 - 65; females were 58% of the total; 75% of clients were unemployed; 37% were illiterate; and 33% had not disclosed their HIV status to their families [13]. A 2002 evaluation of the I-TECH-supported ART facilities in Amhara and Tigray found that 10 - 25% of ART patients did not return for appointments within 30 days of the scheduled date. This finding was part of the impetus to initiate a CM program [14].

### Data collection

A member of the UCSF research team visited the I-TECH offices in Addis Ababa to assess CM expenditures during a 10-day site visit in June 2010. The team member consulted with I-TECH Ethiopia staff, including human resources, treatment program staff, financial administration staff, and the case management coordinator. He also visited two case management sites (Felege Hiwot Hospital and Gondar University Hospital) to gain additional understanding of the operational context and activities of the case management program. Refinement of the cost data collected during the visit was completed over three subsequent months via e-mail and conference call exchanges.

#### Direct services provision

Service delivery personnel costs were collected through the examination of relevant reports generated by I-TECH's QuickBooks^® ^database. The team reviewed records of the I-TECH staff complement for the study period and identified staff associated with the case management program at the study sites. These included Case Managers, Adherence Supporters, and the Case Management Supervisors. We consulted with financial staff to determine relevant salaries, benefits and health insurance costs. Case Manager Supervisors divide their time between two to four sites, and we allocated their compensation expenses across these sites accordingly.

#### Training

Training costs were included in the QuickBooks^® ^database, and these costs were verified through the review of training schedules before and during the study period. Costs included facility rental, refreshments, materials, and associated travel and per diem costs. In the absence of specific relevant data, we assumed that training activities and associated costs would be renewed every three years.

#### Capital purchases

The site visit included meetings with the procurement and operations staff to review the purchase and use of capital goods such as vehicles and office equipment, for the case management program. All capital procurements were amortized over five years of estimated useful life. Donated used computer equipment was assessed at 50% of the market cost of equivalent new equipment.

#### Overhead

Overhead cost data collection was conducted in collaboration with the I-TECH financial management team. The QuickBooks^® ^system does not allocate costs on a by-program basis, so we manually assigned relevant portions of line items to case management from the expenditure reports. Monthly expenditure data were then generated for the study period across all available overhead cost categories, including management personnel costs, curriculum development, office rental, and travel costs. Functions that supported I-TECH activities in ways that were harder to assign to specific programs, such as telecommunications, information technology, and utilities, were allocated to CM in proportion to the CM program's share of I-TECH direct services staff. Headquarter office rental costs were assigned to the CM program based on the proportion of CM office space to all headquarter office space.

#### Outputs

Services volume expressed as "patient-quarters of CM delivered" was tabulated from standardized quarterly reporting forms compiled by Case Manager Supervisors. The number of patient-quarters was estimated using the number of new entries into the CM program the numbers restarting CM after discharge and continuing with the program, and the number exiting each quarter. We assume that entry or exit events were distributed randomly across each respective quarter, and thus occurred on average six weeks (half-way) through the quarter. Thus, the number of patient-quarters in any quarter is: *50% x (New entries + Re-starting clients) + Continuing - 50% x Exits for all reasons*.

The number of patients served cannot be directly obtained from the quarterly report forms. However, a review of log data in Felege Hiwot Hospital (West Amhara Region), Adigrat Hospital (Tigray Region) and Awash Health Center (Afar Region) found that the average duration of CM treatment was 2.78, 2.51 and 2.82 months, respectively. These averages were applied to the other data on patient-quarters of CM sites in the respective regions to derive an estimate of the number of patients served at each site.

The third output measure, "Successful exits", was defined as patients whose barrier to adherence has, in the judgment of the case manager, been resolved, or who have been successfully referred to a community-based organization that affirms that the patient is receiving needed services. The total number of successful exits was obtained from the quarterly report form.

## Results

### Outputs and costs

During the 12-month study period, the I-TECH Ethiopia CM program delivered 4,598 patient-quarters of services in the 14 study sites, or 328 on average [Median per site, 251; sd, 274]. 5,056 patients were served [Median per site: 271; sd, 292]. A total of 1,995 clients successfully exited the program, or 143 per site [Median: 83; sd, 232]. The total value of resources consumed by the CM program across the 14 sites, including the amortized costs of capital items and training, and allocated overhead expenses, was $167,457, or an average of $11,961 per site [Median: $11,268; sd, $3,965]. See Table 1.

**Table 1:** I-TECH CM program outputs, total costs and unit costs at 14 ART delivery sites.

		**CM program outputs**			**Cost**	**Unit costs**		
**Province**	**ART site**	Patient-Quarters of CM	Patients served^1^	Successful exits	Expenditures for Case Management	Cost per Patient-quarter of CM	Cost per person served	Cost per successful completion
	Debre Markos	529	570	89	$10,419	$20	$18	$117
	Woldia	234	253	76	$12,465	$53	$49	$164
	Gondar	269	290	100	$16,007	$60	$55	$160
**Amhara**	Debire Tabor	85	91	18	$12,136	$144	$133	$674
	Debre Berhan	168	181	128	$7,577	$45	$42	$59
	Dessie	435	469	159	$19,330	$44	$41	$122
	Felege Hiwot	1,146	1,237	933	$18,775	$16	$15	$20
	Axum	270	323	45	$8,519	$32	$26	$189
	Humera	153	183	129	$14,603	$95	$80	$113
**Tigray**	Alamata	199	238	142	$9,335	$10	$39	$66
	Mekelle	195	232	42	$11,667	$60	$50	$278
	Adigrat	105	125	49	$9,468	$90	$75	$193
	Awash	525	558	56	$10,869	$21	$19	$194
**Afar**	Dubti	287	305	29	$6,288	$22	$21	$217
**Total**		**4,598**	**5,056**	**1,995**	**$167,457**	**N/a**	**N/a**	**N/a**
**Mean per site**:		**328**	**361**	**143**	**$11,961**	**$51**	**$48**	**$183**
	Standard Dev.	274	292	232	$3,965	$38	$32	$157
	Median:	251	271	83	$11,268	$45	$41	$162
					**Mean per-patient cost**	**$36**	**$33**	**$84**

Data from October 2008 - September 2009. ^1. ^Patients served derived from caseload log counts from hospitals in Felege Hiwot, Adigrat and Awash. Patients served were estimated for the other facilities in each province based on the ratio of patients served to patient-quarters provided in the hospital in the province for which log data were tabulated.

The average cost per patient-quarter of CM services was $36 [Median: $45; sd, $38]. The average cost per patient served was $33 [Median: $41; sd, $32] and the average cost per client who successfully exited the CM program was $84 [Median: $162; sd, $157]. The rural hospital at Debire Tabor had the highest unit costs ($114 per patient-quarter and $674 per successful exit), the result of roughly average total cost distributed over the smallest CM caseload. Felege Hiwot referral hospital had the lowest unit costs ($16 per patient-quarter and $20 per successful exit), accounting for 24.9% of CM patient-quarters delivered and 46.7% of all successful exits.

**Figure 1:**
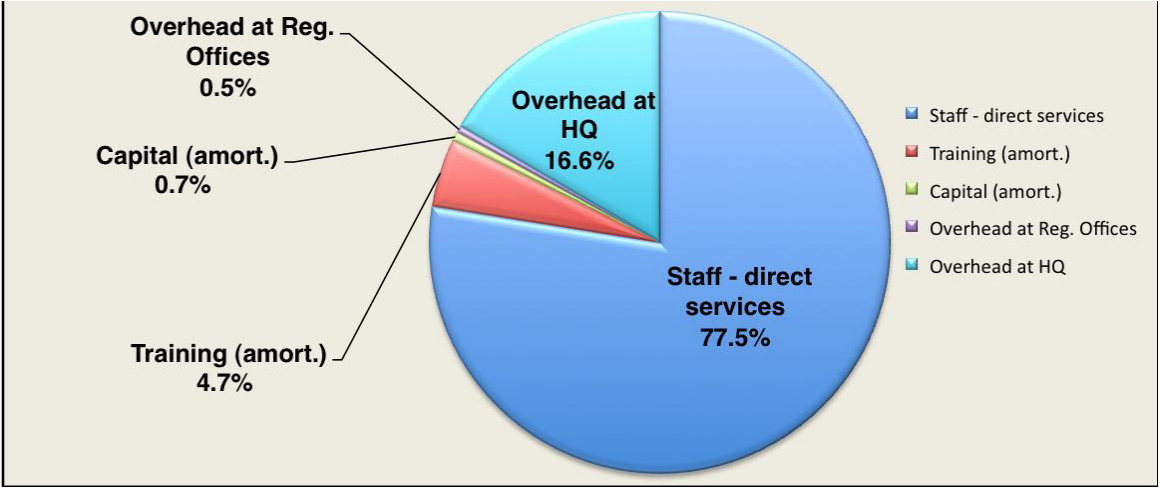
**Program costs by major component in 14 ART-provision sites in Amhara, Tigray and Afar provinces**. Data from October 2008 through September 2009.

Compensation for personnel delivering CM services and their immediate supervisors accounted for 77.5% of spending; 16.6% supported overhead costs at I-TECH headquarters in Addis Ababa; 4.7% was due to the amortized cost of training; 0.7% was for the amortized cost of capital goods; and 0.5% supported overhead at the regional I-TECH offices (Figure 1). Overhead costs at headquarters in Addis Ababa consisted of contractual services for curriculum and guidelines development (33.9%); administration (27.0%), telecommunications and I.T. (17.2%); rent (10.6%); utilities (2.0%) and miscellaneous expenses such as printing and travel (9.2%). See Figure 1.

### Variations by scale

**Figure 2:**
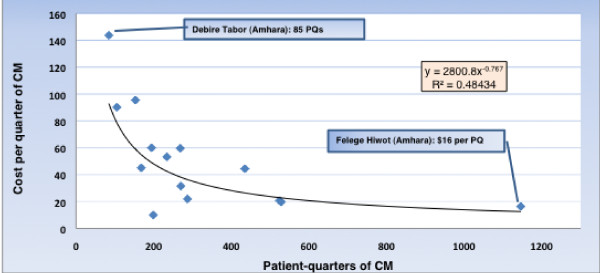
**Variation in unit cost: Cost per patient-quarter of CM services provided**. Data from October 2008 through September, 2009; (n = 14 I-TECH supported ART sites).

**Figure 3:**
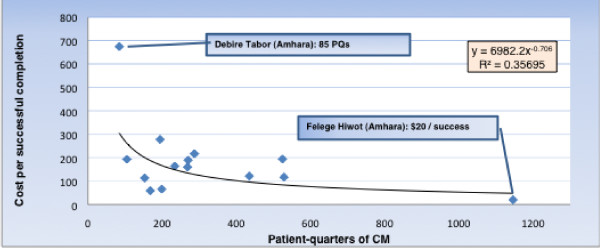
**Variation in unit cost: Cost per successful exit from CM services**. Data from October 2008 through September, 2009; (n = 14 I-TECH supported ART sites).

In bivariate analyses, scale was inversely associated with unit cost for both patient-quarters and successful exits, with R^2 ^of 0.484 and 0.357, respectively. (See Figures 2 and 3).

The cost per successfully exited client and the cost per patient quarter of CM have a correlation of 0.725 (R^2^, 0.53). The correlation between the number of successful exits and the number of patient-quarters of CM delivered is 0.86 (R^2^, 0.74).

We also compared unit cost with the ratio of the volume of services at each site to the number of full-time equivalent staff delivering direct services. We found that the correlation between the two was negative; that is, the sites with higher volumes of services per staff member tended to have lower unit costs (r = -0.49 and -0.68 for cost per successful exits and cost per patient-quarters of CM delivered, respectively).

#### Potential for greater efficiency

As shown in Table 1 and in Figures 2 and 3, there is substantial variation in unit costs among the 14 study sites. This may suggest the potential for greater efficiency in the more expensive sites. If sites with unit costs at the 75^th ^percentile or higher had the average unit costs of those under the 75^th ^percentile, the savings could fund additional services that would increase the number of patient-quarters of CM by 23.7%, and the number of successful exits by 22.8%. If it were possible for the program as a whole to emulate the performance of Felege Hiwot, a 2.2-fold increase in patient-quarters of CM services and a 4.2-fold increase in successful exits could be sustained by the $167,457 spent during the study period.

## Discussion

In this study examining the costs and outputs of 14 I-TECH sponsored HIV treatment case management sites in Ethiopia, we found that the I-TECH Ethiopia program delivered 4,589 patient-quarters of services, served 5,056 patients and 1,995 successful exits at a cost of $167,457 over 12 months, or $36 per patient-quarter, $33 per patient served, and $84 per successful exit. Unit costs varied inversely with scale across sites. These results suggest that higher services volume may be an important determinant of higher efficiency for CM. However, there are limitations to what program managers can do to increase ART and CM uptake, as this depends importantly on HIV prevalence, population density, and access issues which may be outside of their control. However, we also found strong negative correlation between client-staff ratios and unit costs. This indicates that observed economies of scale are not only due to the spreading of fixed costs over larger services volumes, but that higher service volumes are associated with higher staff productivity. How the relationship between volume and productivity is mediated is not clear from our data. Personnel costs are conventionally classified as variable costs. However, a case manager may in the short run be a fixed cost in that "one" is the indivisible lowest unit that can be assigned even to a low-volume site. It is possible that efficiency would be increased, even absent greater volume if, at sites with higher costs and lower volumes, case managers were able to divide their time between CM and other HIV-related services. Alternatively, if there are sites with under-utilized case manger staff time, it might be possible to intensify CM services to clients and potentially generate a larger number of successful exits with existing staff levels. Some degree of flexibility and adaptability of the CM model to local conditions may be essential for efficient service delivery. Indeed, at some sites case managers have started to assume limited responsibility for other services to HIV inpatients.

A significant limitation of this analysis is that it was not always possible to quantify the portion of administrative staff time and other overhead resources that would have been needed in the absence of the CM program, i.e., the incremental opportunity cost of the overhead inputs. We estimated the average cost of general overhead inputs by allocating them to CM according to the proportion that CM service providers constitute of all I-TECH service providers supported by these overhead functions. A second limitation is that the definition of "successful exits" from the program was not operationalized consistently across the study sites, nor was it linked to quantified improvements in adherence. However, the high correlations between service volumes and "successful exits," and between the unit costs of these two outputs measures, suggest that despite variation across sites in its precise operational definition, the number of "successful exits" appears to be an outcome predominantly determined by the extent of services. Thus, varied definitions may still represent aspects of the same underlying process.

Cost analyses have a variety of important management and budget projection uses. However, since they do not evaluate health outcomes, they do not provide the guidance for program funding or resource allocation decisions conferred by cost-effectiveness analyses. A definitive analysis of the cost-effectiveness of improving adherence would entail a comparative assessment of an array of effective measures to improve adherence. These include eliminating ART co-payments (in settings that have them), payments for drugs to treat opportunistic infections, improved personnel training, and providing meals and reimbursing for transportation for participants [11]. They might also include use of electronic devices for medication use monitoring, other economic incentives, and cell phone-based supervision [15]. A recent review of interventions to increase adherence to ART in sub-Saharan Africa found that some methods appear to have benefit but that these benefits may be limited and temporary, or be confined to specific settings or the precise intervention content. In addition many of the results depended on observational studies rather than controlled trials [16]. Thus, completion of a technically sophisticated analysis may be years away. Pending funding for randomized, multi-arm trials that document the costs and benefits of a range of adherence promotion strategies, a first step would be a cost-effectiveness analysis of a CM program versus a standard ART program without any adherence intervention. Lacking strong evidence on which candidate methods are likely to be most cost-effective, program managers should also be encouraged to try methods they deem appropriate for their respective settings, and to document the costs and resulting changes in adherence or health status.

Absent a cost-effectiveness assessment, it is nevertheless useful to place our unit cost estimates in context. ART has recently been estimated to cost $643 per patient-year in Ethiopia [17]. Discounting at 3% per annum, and assuming 12 ART-years, the lifetime cost of ART is $6,400. The CM program thus adds 0.52% ($33/$6,400) to the lifetime cost of treatment for those patients receiving CM. Let us assume, perhaps pessimistically, that the CM program has no effect on 90% of those classified as having had a "successful exit" and that the remaining 10% are returned to normal adherence. The $840 ($84/10%) per CM client restored to normal adherence would thus add 13.1% to the lifetime cost of ART ($840/$6,400) per successful exit; or the equivalent of an additional 16 months of ART ($840/$643) for the patients receiving CM. This rough calculation does not account for changes in treatment costs resulting from greater adherence, or for the potential reduction in transmission to partners including the transmission of drug-resistant strains of HIV.

The evaluation of incremental cost-effectiveness may not form a sufficient basis for deciding whether to invest in CM services. Once patients have been accepted for treatment, there is an ethical obligation to make reasonable efforts to ensure that they benefit from those services. Yet, this perspective begs the question of what constitutes a "reasonable" additional effort, especially in resource-constrained countries such as Ethiopia. Program managers will not invest unlimited resources in a small number of poorly adhering patients, at the expense of expanded access to ART or less intensive but successful CM efforts. Ultimately, what is deemed "reasonable" depends on a wide range of factors that may be best decided by local program managers. Nevertheless, this decision should be informed by an understanding of the cost-effectiveness of additional investments in CM. Finally, the variation in unit costs reported here suggest that there may be opportunities to identify staffing patterns that increase overall program efficiency, thus freeing increasingly constrained HIV resources with no additional investment.

## Conclusion

Case management for ART patients in Ethiopia who are at risk for poor adherence costs an average of $33 per patient served, or $84 per patient who successfully exits the program. The data reported in this study reflect wide variation in unit costs among the study sites and suggest that high patient volume may be a major determinant of CM program efficiency. The observed variations in unit costs also indicate that there may be opportunities to identify staffing patterns that increase overall program efficiency.

## List of abbreviations used

ART: Antiretroviral therapy; CM: Case management; FY: Fiscal Year; GFATM: Global Fund to Fight AIDS, Tuberculosis and Malaria; I-TECH: International Training and Education Center for Health; PEPFAR: United States President's Emergency Plan for AIDS Relief.

## Competing interests

The authors declare that they have no competing interests.

## Authors' contributions

EM led the process of research design, data analysis and interpretation, and the manuscript drafting and revision effort. SK carried out data collection and cleaning, data analysis and interpretation, manuscript drafting and editing. IA carried out data collection and cleaning, data analysis and interpretation and manuscript editing. GF and BG carried out data analysis and interpretation and manuscript editing. TH participated in research design, data analysis and interpretation, manuscript drafting and editing. JGK and MR participated in research design, data interpretation and manuscript editing. All authors read and approved the final manuscript.
